# Verbal Lie Detection: Its Past, Present and Future

**DOI:** 10.3390/brainsci12121644

**Published:** 2022-12-01

**Authors:** Aldert Vrij, Pär Anders Granhag, Tzachi Ashkenazi, Giorgio Ganis, Sharon Leal, Ronald P. Fisher

**Affiliations:** 1 Department of Psychology, University of Portsmouth, Portsmouth PO1 2DY, UK; 2 Department of Psychology, University of Gothenburg, 405 30 Gothenburg, Sweden; 3 Department of Criminology, Bar Ilan University, Ramat Gan 5290002, Israel; 4 Department of Criminology, Ashkelon Academic College, Ashkelon 78211, Israel; 5 School of Psychology, Brain Research and Imaging Centre, University of Plymouth, Plymouth PL4 8AA, UK; 6 Department of Psychology, Florida International University, Miami, FL 33199, USA

**Keywords:** verbal lie detection, fMRI lie detection, criteria-based content analysis, reality monitoring, scientific content analysis, cognitive credibility assessment, reality interviewing, strategic use of evidence

## Abstract

This article provides an overview of verbal lie detection research. This type of research began in the 1970s with examining the relationship between deception and specific words. We briefly review this initial research. In the late 1980s, Criteria-Based Content Analysis (CBCA) emerged, a veracity assessment tool containing a list of verbal criteria. This was followed by Reality Monitoring (RM) and Scientific Content Analysis (SCAN), two other veracity assessment tools that contain lists of verbal criteria. We discuss their contents, theoretical rationales, and ability to identify truths and lies. We also discuss similarities and differences between CBCA, RM, and SCAN. In the mid 2000s, ‘Interviewing to deception’ emerged, with the goal of developing specific interview protocols aimed at enhancing or eliciting verbal veracity cues. We outline the four most widely researched interview protocols to date: the Strategic Use of Evidence (SUE), Verifiability Approach (VA), Cognitive Credibility Assessment (CCA), and Reality Interviewing (RI). We briefly discuss the working of these protocols, their theoretical rationales and empirical support, as well as the similarities and differences between them. We conclude this article with elaborating on how neuroscientists can inform and improve verbal lie detection.

## 1. Introduction

The four most common ways to detect deception is via observing nonverbal behaviour, analysing speech content, measuring physiological responses, or measuring brain activity. In terms of research, physiological lie detection came first in the 1930s [1], followed by nonverbal lie detection in the 1950s [2], verbal lie detection in the 1970s [3], and brain activity lie detection in the 1980s [4,5,6]. 

This article focusses on verbal lie detection, which we think is particularly valuable. Lie detection through measuring physiological reactions or brain activity typically requires hooking up interviewees to machines to measure such responses, making physiological reactions relatively intrusive and difficult to use. When considering verbal and nonverbal lie detection, only verbal lie detection may lead to conclusive evidence that can be used in courts (e.g., contradicting facts), and investigators elicit longer statements from interviewees when applying verbal compared with nonverbal lie detection [7] because longer statements are required for verbal lie detection [8]. This eliciting-information aspect of verbal lie detection is beneficial because eliciting information is an important aim of interviewing [9].

## 2. Initial Verbal Lie Detection Research

Initial verbal lie detection research started in the late 1970s [3,10] and focused on individual words such as (i) self-references (‘I’ or ‘We’), (ii) negative emotions (‘hate’, ‘worried’, ‘nervous’ or ‘sad’), (iii) generalising terms (‘always’, ‘never’), (iv) lexical diversity (percentage of distinct words divided by total number of words), and (v) exclusive words (‘without’, ‘except’ or ‘but’) [11,12]. This type of lie detection is still popular because such words can be detected automatically with computer programs [11]. A meta-analysis [11] showed that lie tellers made more generalising terms than truth tellers (g_u_’s = −0.37) but this was based on only four studies, which means it should be interpreted with caution. Exclusive words were also related to deception and examined enough times (n = 20) to obtain reliable results: lie tellers used more exclusive words than truth tellers (g_u_ = 0.31). The other three cues (self-references, negative emotions, and lexical diversity) were examined at least 22 times but none of the cues were related to deception (g_u_’s ranged from −0.18 to 0.14). In other words, verbal lie detection research examining individual words revealed some veracity indicators.

## 3. From the 1980s Onwards: Clusters of Verbal Cues

From the 1950s onwards, scholars from Sweden [13] and Germany [14,15] examined verbal criteria thought to be related to the credibility of child witnesses’ testimonies in trials for sexual offences. It is often difficult to determine the facts in an alleged sexual abuse case. Frequently, the alleged victim and the defendant give contradictory testimony and there is no independent evidence available to determine an objective version of events. This makes the perceived credibility of the alleged victim and defendant important. The alleged victim is in a disadvantageous position if s/he is a child, because adults tend to mistrust children’s statements [16].

The German scholars Günter Köhnken and Max Steller refined the available criteria and integrated them into a formal list of 19 verbal criteria, which they published in English in the late 1980s [17,18]. In a procedure called Criteria-Based Content Analysis (CBCA), experts assess the presence of each of the 19 criteria in a written statement. The 19 CBCA criteria are all cues to truthfulness and are thought to be more frequently present in truthful than deceptive statements [19]. CBCA is part of a wider veracity assessment procedure called Statement Validity Assessment (SVA) that consists of four stages: (i) a case-file analysis to gain insight into the case; (ii) a semi-structured interview to obtain a statement from the interviewee; (iii) CBCA that systematically assesses the quality of a statement; and (iv) an evaluation of the CBCA outcome via a set of questions (Validity Checklist) [20,21,22]. 

The first 13 CBCA criteria are cognitive criteria. According to the CBCA rationale, these criteria are more likely to occur in truthful than in deceptive statements because lie tellers find it typically too difficult to fabricate them [23]. The cognitive criteria include reporting information in non-chronological order (Criterion 2, unstructured production); the total amount of information reported (Criterion 3, total details); mentioning where (location) and when (time) events took place (Criterion 4, contextual embeddings); and reporting experiences that were unexpected (Criterion 7, unexpected complications). Lie tellers are less likely than truth tellers to report the remaining criteria for motivational reasons: lie tellers believe that including these criteria in their statement make them sound suspicious [23]. An example of such a suspicious-sounding criterion is making corrections without being prompted by the interviewer (Criterion 14, Spontaneous corrections). 

Different ways are used to code the presence of CBCA criteria. Sometimes 3-point Likert scales are used (a criterion is never, occasionally, or often present); sometimes 7-point Likert scales are used (a criterion is [1] not at all present to [7] very often present); and sometimes the number of times each criterion occurred (frequency of occurrence) is counted [12]. Whether using different coding schemes affects the diagnostic value of CBCA has, to our knowledge, never been examined. 

CBCA has been widely researched. One meta-analysis [24] included 39 studies analysing adult statements and another meta-analysis [25] included 22 studies analysing child statements. Truth tellers obtained higher total CBCA scores (summation of the individual criteria) than lie tellers in adults (*d* = 0.55) and in children (*d* = 0.78). The total CBCA scores discriminated truth tellers and lie tellers better than each of the individual criteria in both adults and children. The finding that examining clusters of cues yields better lie detection results than examining individual cues is a typical finding in deception research [26,27]. In both adults’ and children’s statements, the cognitive criteria were better veracity indicators (average *d*-scores were *d* = 0.29 in adults and *d* = 0.42 in children) than the motivational criteria (average *d*-scores were *d* = 0.13 in adults and *d* = 0.19 in children). 

CBCA has become a game-changer in verbal lie detection in several ways. First, CBCA experts examine a cluster of cues rather than the earlier practice of examining individual cues. Second, CBCA is a standardised veracity assessment method unlike the unstandardised verbal (or nonverbal) lie detection methods that were used before (After the introduction of CBCA, a standardised protocol to analyse nonverbal behaviours emerged: the Behavior Analysis Interview (BAI). Although the initial results were promising [28], they have not been replicated since [29,30,31].) Standardisation means that CBCA experts always examine the same 19 verbal cues in every single statement they analyse. In contrast, until the arrival of CBCA, verbal and nonverbal lie detection was carried out randomly—experts did not have fixed lists of cues they examined, nor could they tell beforehand which cue they would attend to. A prime example of this is the leakage hypothesis, popular in nonverbal lie detection [32,33,34]. Lie tellers are supposed to leak nonverbal cues to deception, but what these leaking nonverbal cues are remains undefined. The (unspecified) cues could literally be any behaviour. Experts did not predict beforehand which cues may reveal leakage, and when analysing footage, different experts may highlight different cues. 

A second verbal veracity assessment tool based on a cluster of verbal criteria emerged in the early 1990s: Reality Monitoring (RM: [35,36,37]. The RM lie detection tool is based on the work of Marcia Johnson and Carol Raye who published in 1981 their seminal paper about memory characteristics [38] (see also [39,40]). They argued that memories of real experiences differ from memories about imagined events. Memories of real experiences are obtained through perceptual processes. Memories of real experiences are therefore likely to contain *sensory information*—details of vision, sound, smell, taste, or touch. These experiences took place in space and time and therefore contain *contextual information*, which consists of spatial details (details about where the event took place but also where objects and people were situated in relation to each other) and temporal details (details about the time order of events and the duration of events). In contrast, memories about imagined events are derived from an internal source and are therefore likely to contain *cognitive operations*, such as thoughts and reasonings (‘I must have had my coat on, as it was very cold that night’). 

In RM deception research, it is assumed that truths are recollections of experienced events whereas lies are recollections of imagined (simulated) events [41,42,43]. RM has also been widely researched and the most recent meta-analysis included 40 studies [44]. Truth tellers obtained a higher RM score (total score of the individual criteria) than lie tellers (*d* = 0.55). Regarding the individual criteria, their relationship with deception was always weaker than the relationship between deception and the total RM score. These relationships ranged between *d* = 0.25 for spatial information to *d* = 0.51 for temporal information. 

A final verbal veracity assessment tool that examines a cluster of verbal criteria is Scientific Content Analysis or SCAN [45]. A definite list of the observed criteria does not exist, and different SCAN users use different combinations of about 12 criteria [46,47,48]. They include cues of truthfulness (cues that truth tellers are thought to report more than lie tellers) and cues of deceit (cues that lie tellers are thought to report more than truth tellers). Examples of cues of truthfulness are denials of allegations and the use of pronouns (‘I’, ‘my’, ‘we’, ‘us’). Examples of cues of deceit are text bridges which imply missing information (words such as ‘finally’, ‘later on’, and ‘sometimes after’) and change in language (describing in a single statement a car as ‘the car’, ‘the vehicle’, and ‘the dark coloured car’).

No theoretical rationale is given why truth tellers and lie tellers are expected to differ on the SCAN criteria, and there is not much research that has been carried out with SCAN. The developer, Avinoam Sapir, never reported any formal empirical test of the tool, but SCAN supporters often refer to a study by Driscoll [47] which showed very good results (about 85% total accuracy). This was a field study in which the statements of real criminal suspects were analysed. The problem was that the ground truth was mostly unknown; that is, it was not known which suspects were telling the truth and which suspects were lying [12]. Experimental laboratory research into SCAN in which the ground truth is known has not shown any evidence that SCAN works as a lie detection tool [46,49,50].

## 4. Comparing CBCA, RM and SCAN

Table 1 summarises the aspects of the CBCA, RM, and SCAN tools that we believe are important when comparing the three tools. All three use a cluster of variables to assess veracity, but only CBCA and RM do this in a standardised way. That is, in evaluating each statement, different CBCA and RM experts use the same list of criteria and use the same procedure in assessing veracity based on these criteria (by creating total CBCA and RM scores). In contrast, SCAN is not standardised. An official list of SCAN criteria does not exist although there seems to be agreement amongst SCAN users about which 12 criteria to use. No standard procedure exists, however, of how to assess veracity based on these 12 criteria. SCAN experts do not create a total score but highlight the criteria they believe are indicative of truth or lie in each statement. The criteria they highlight depend on the statement and on the SCAN expert [51]. Unsurprisingly, when assessing statements, inter-agreement among SCAN experts is typically low [48].

Total CBCA and RM scores appear to be diagnostic veracity indicators. When comparing these two methods—which is only possible in adult statements because of the absence of RM data in child statements—they appear to be equally diagnostic. Furthermore, CBCA and RM have been examined across many labs which contributes to the robustness of the results. The CBCA meta-analysis with child statements included more than 15 different labs, whereas the CBCA meta-analysis with adult statements and the RM meta-analysis each included more than 20 different labs. SCAN does not appear to be a valid veracity assessment tool. Although SCAN is not widely researched, it has been carried out in several labs, predominantly by Bogaard et al. [46,49,52] but also by [47,48,50,53].

Almost all psychology theory and research originated in Western, educated, industrialised, rich, and democratic (WEIRD) nations and lie detection is not an exception. Verbal deception research in non-WEIRD countries is scarce but relevant because cross-cultural differences in verbal cues to deceit exist [54,55,56]. We are not aware of CBCA and RM research carried out in non-WEIRD countries and the CBCA and RM meta-analyses published to date do not make a WEIRD vs non-WEIRD comparison. However, it has been suggested that some CBCA criteria may be sensitive to cultural differences [57]. 

Practitioners are more likely to use SCAN than either CBCA or RM. Practitioners across the world use this tool, including law enforcement personnel, military agencies, and secret services [50]. RM, to our knowledge, is not used by practitioners but is popular amongst researchers. SVA assessments (of which CBCA is part) are made by legal psychologists in alleged sexual abuse cases in several Western European countries and in Canada and the SVA verdicts are accepted as evidence in criminal courts in those countries [12,58]. 

SVA assessments are not identical to CBCA assessments [20]. The problem is that CBCA scores are affected by factors other than veracity. To give two examples: CBCA scores are affected by the age of the interviewee (older children provide higher quality statements than younger children and thus obtain higher CBCA scores) [59,60] and by the quality of the interview (a well-conducted interview elicits more information than a poorly conducted interview and thus results in higher CBCA scores) [61,62]. 

In experimental CBCA research, groups of truth tellers and lie tellers are compared and these groups are similar except for their veracity status. In other words, the external factors that could influence CBCA scores are controlled for. In real life, individual cases are examined, and CBCA experts need to take the impact of external factors into account, such as age and quality of the interview, when making veracity assessments. This is the purpose of the Validity Checklist procedure, the fourth phase of SVA. The Validity Checklist procedure is more subjective and less formalised than the CBCA procedure [18,19]. When examining the use of the Validity Checklist in Sweden, different experts sometimes drew different conclusions about the impact of Validity Checklist factors on children’s statements [63]. If two experts disagree about the veracity of a statement in a German criminal case, the most likely reason for the disagreement is that they have a different view about the impact of the Validity Checklist factors on that statement (Vrij [12] citing personal communication with Köhnken in 1997). In addition, in CBCA research, equal weight is given to each criterion when calculating a total CBCA score, whereas this is often not the case in real life [20]. In other words, although researchers treat CBCA as a quantitative method, this is not the case in real-life practice [20].

Both CBCA and SCAN training courses are multiple-day courses. CBCA is more difficult to learn and to use than SCAN because CBCA is part of the wider SVA procedure [12,58]. To make someone familiar with the entire SVA procedure, [23] recommends a 3-week training course.

Researchers are more likely to examine RM than either CBCA or SCAN. At least two factors contribute to this. First, RM coding is relatively easy to learn and use [12,43]. That is, trainees feel comfortable about how to conduct RM coding after a few hours of training. Second, most research deals with interviewing lie tellers and truth tellers about alleged experiences. Those experiences are derived from sensory details (vision, sound, smell, taste, and touch) and since the experiences happened in time and space, they also include contextual details (time and space). The RM list of criteria includes these five sensory categories and two contextual details categories, which means that all possible details that describe experiences are covered by RM coding. In other words, RM gives a complete picture of what interviewees recall. The CBCA and (generally used) SCAN criteria include the two contextual details criteria, but the other criteria are not categorised based on type of sensory information. Instead, they are related to specific types of content. All sensory information that does not match these content criteria will be omitted in CBCA and SCAN coding. In other words, CBCA and SCAN do not give a complete picture of what an interviewee recalls.

According to the CBCA rationale, lie tellers provide less information than truth tellers because lie tellers are unable to fabricate all sorts of detail (so-labelled cognitive criteria). This inability to fabricate details is nowadays still mentioned by deception researchers. However, researchers nowadays also mention an additional reason: lie tellers are unwilling to report information because they wish to keep their stories simple, as we explain in the next ‘Interviewing to detect deception’ section. 

Note that an inability to report details and an unwillingness to do so lead to the same outcome—lie tellers report fewer details than truth tellers. It is difficult to determine what it is causing lie tellers to provide fewer details than truth tellers, whether this is because of the lie tellers’ inability to report information, their unwillingness to do so, or a mixture of the two. Research has revealed that unwillingness to report information on its own can result in lie tellers reporting fewer details than truth tellers. In one experiment, truth tellers and lie tellers were sent on the same mission and were asked to report their experiences in a subsequent interview [64]. Lie tellers were instructed to lie about the reason for carrying out the mission, not about what they did. In the interview, no questions were asked about why the interviewees carried out their mission; they were just asked to tell what they did. Lie tellers could thus report their missions truthfully and did not have to fabricate any details. Yet they reported fewer details than truth tellers. This cannot be explained by the lie tellers’ inability to fabricate details. In another experiment, truth tellers and lie tellers carried out the same mission [65]. Truth tellers were instructed to report all details truthfully; lie tellers were instructed to report most details truthfully, except one aspect of the mission, which they were instructed to omit (omission lie). Lie tellers reported fewer details than truth tellers when comparing the parts of the mission that both truth tellers and lie tellers reported truthfully. This finding also cannot be explained by the lie tellers’ inability to fabricate details but there was evidence that it was associated with the lie tellers’ unwillingness to report information. That is, lie tellers more than truth tellers reported keeping their stories simple and this strategy was negatively associated with the number of details provided. 

The RM rationale is that truth tellers report more sensory and contextual details than lie tellers because truth tellers have experienced the event, whereas lie tellers have only imagined the event. This seems to refer (implicitly) to the lie tellers’ *inability* to report information—they have not experienced sensory and contextual details, so they do not report them—rather than to their *unwillingness* to do so. However, instead of fabricating a story (result of imagination), lie tellers more often tell embedded lies in which large parts are true [66]. Lie tellers also report fewer sensory and contextual details than truth tellers in embedded lies, which is difficult to explain with the RM rationale. We believe that the merit of RM lies in the quality of detail it examines (sensory and contextual details) as researchers still widely examine such details to date. 

We do not know what the rationale is behind SCAN because SCAN users have never provided such a rationale. Since the SCAN instrument does not seem to differentiate truth tellers from lie tellers, we do not find it necessary to speculate on the possible rationale. 

The RM and SCAN tools do not include interview protocols. SCAN takes this to the extreme. Interviewees are instructed to write down their activities during a specific period in as much detail as possible. Interviewers are not present when the interviewees write down their experiences, so that they cannot influence the statement. The SCAN intentions are good because poor interviewing can impair the ability to detect deceit [7,8,67]. However, research has shown that verbal differences between truth tellers and lie tellers in unprompted recalls are faint and unreliable [68] and that the opportunity to distinguish between truth tellers and lie tellers in unprompted recalls is just above chance level [69]. The limitation of the SCAN procedure is that it ignores that truth tellers do not recall all information they know in an unprompted recall [70]. In addition, practitioners find obtaining written statements problematic because written statements are not common in police interviews [51]. CBCA is part of SVA which includes an interview protocol (Phase 2). However, this interview protocol is not tailored to lie detection. It advises to interview according to good practice interview principles but does not discuss which specific questions or instructions are required to enhance the ability to detect deceit.

Inspired by [68] meta-analysis showing that verbal cues to deceit in unprompted recalls are faint and unreliable, verbal lie detection researchers started to examine whether verbal cues could be elicited or whether already existing verbal differences could be further enhanced using specific interview protocols. Four interview protocols have emerged since then and we will discuss them in the next section. They all assume that specific questions or specific instructions will lead to different verbal responses by truth tellers and lie tellers, which subsequently facilitates lie detection. The idea that the interview protocol matters in truth/lie detection is not new. It has been acknowledged in physiological lie detection from the moment it emerged in the 1930s. The debate in that research domain is heated [12,71]. It is not about the ability of the polygraph to distinguish between truth tellers and lie tellers because all researchers involved in the debate are using the same machine and examine the same physiological responses. The debate is about which interview protocol to use to obtain reliable differences between truth tellers and lie tellers [72,73]. A heated debate amongst verbal deception researchers like the one in the polygraph world is absent because the verbal interview protocols are not in competition with each other. Which protocol to use depends on the situation as we explain in the next section.

## 5. Interviewing to Detect Deception

Verbal lie detection made the next step in its development in the mid-2000s when the first ‘interviewing to detect deception’ articles emerged [74,75]. Scholars started to design interview techniques aimed at enhancing or eliciting cues to truthfulness or deceit. The starting point of this line of research was that lie tellers and truth tellers enter interviews with different mental states [76]. Lie tellers have unique knowledge, which, if recognised by the interviewer, makes it obvious that they are lying. Lie tellers’ main concern is to ensure that the interviewer does not gain that knowledge. Innocent suspects face the opposite problem, fearing that the interviewer will not learn or believe what they know. These different mental states result in different strategies used by lie tellers and truth tellers in interviews to sound convincing [77,78,79]. 

One popular strategy used by lie tellers is to avoid providing self-incriminating information [76]. The reason for this strategy is obvious: providing incriminating information will give the lie away. A second popular strategy amongst lie tellers is to keep their stories simple [77]. They do this for several reasons. First, the more information they provide, the more likely it will be that they will provide leads to investigators that gives their lies away [76,80]. Second, lie tellers may expect to be interviewed repeatedly about an event in which case they should not contradict what they have said before. Keeping their initial story simple reduces the likelihood of contradictions occurring in subsequent interviews. Third, although truth tellers believe that their innocence shines through [81,82], lie tellers often take their credibility less for granted than truth tellers [68]. Therefore, lie tellers are more inclined than truth tellers to engage themselves in tasks other than storytelling, such as controlling their demeanour so that they appear honest to the interviewer [83] and monitoring the interviewer’s reactions to assess whether they appear to be convincing [84]. Keeping stories simple is one way to cope with the additional cognitive demand of multi-tasking. 

Truth tellers are inclined to be cooperative and forthcoming [76,77]. However, truth tellers never spontaneously report all information in the first instance [70]. They may have the wrong expectations about how much information they should report, they may lack the motivation to report all the information they can remember, or they may find it difficult to recall all relevant information stored in their memory.

Four interview protocols have been developed to date aimed at exploiting the different strategies truth tellers and lie tellers use: Cognitive Credibility Assessment [85,86,87], Reality Interviewing (RI, [88,89]), Strategic Use of Evidence (SUE, [90,91,92]), and the Verifiability Approach (VA, [93,94,95]). SUE and VA are evidence-based interview protocols: interviewers should possess independent evidence (SUE) or truth tellers must be able to report such independent evidence during the interview (VA) to use them. They focus on the ‘avoiding reporting incriminating evidence’ strategy lie tellers employ and are designed to exploit differences between lie tellers and truth tellers in being evasive or forthcoming in reporting critical evidence. They compare the statements with the (potentially) available evidence (e.g., statement – evidence inconsistency). CCA and RI are statement-quality-based interview protocols: They do not require independent evidence to be available but focus on the quality of the statement. They focus on lie tellers’ ‘keeping-stories-simple’ strategy and are designed to exploit differences between lie tellers and truth tellers in being reluctant or forthcoming when reporting information in general. They focus on the richness of details (e.g., complications). 

### 5.1. Strategic Use of Evidence

According to the SUE rationale, lie tellers’ strategy to avoiding incriminating evidence results in using avoidance strategies (e.g., in a free recall to avoid mentioning that they were at a certain place at a certain time) or denial strategies (e.g., denying having been at a certain place at a certain time when directly asked) [76,90,96]. When investigators possess critical and possibly incriminating background information (independent evidence), they can exploit lie tellers’ avoidance and denial strategies by introducing the available evidence during the interview in a strategic manner. According to the Evidence Framing Matrix (EFM), the pieces of evidence vary in terms of *strength of the source* (from weak to strong) and *degree of precision* (from low to high). Reference [91] found that revealing the evidence in a stepwise manner moving from the most indirect form of framing (weak source/low specificity, e.g., ‘We have information telling us that you recently visited the central station’) to the most direct form of framing (strong source/high specificity, e.g., ‘We have CCTV footage showing that you collected a package from a deposit box at the central station, ground floor level, on the 24th of August at 7.30 p.m.’) to be most successful in obtaining veracity differences. When questions are asked in the most indirect form of framing, the answers of the avoidant/denying lie tellers will be less consistent with the available evidence (e.g., admitting visiting the central station but no admission yet about when and about collecting a package from the deposit box) than the answers of the forthcoming truth tellers (statement-evidence inconsistency [92]). In addition, when more direct forms of framing questions are asked, lie tellers may start to realise that the interviewer may hold incriminating evidence against them. Lie tellers are then inclined to change their statement and try to provide an innocent explanation for this evidence. As a result, lie tellers will show less overlap between different parts of a statement than truth tellers (within-statements inconsistency). See [96] for a recent overview of the SUE technique. 

The SUE technique has been widely researched with over 20 publications known to us. A meta-analysis of eight SUE studies showed a very large veracity effect in statement—evidence consistency (*d* = 1.06), the only verbal cue examined in that meta-analysis [92].

### 5.2. Verifiability Approach

The idea behind the Verifiability Approach [94,95] is that lie tellers have a dilemma to solve. The subjective belief amongst people is that deceptive statements lack detail [12]. Indeed, the richer an account is perceived to be in detail, the more likely it is to be believed [97]. Lie tellers are therefore motivated to provide many details in their attempts to come across as sincere. However, at the same time, lie tellers prefer to avoid mentioning too many details out of fear that investigators will check such details, which could subsequently give the lie away [98]. A strategy that incorporates both goals is to provide many details, but to avoid reporting details that can be verified. Details that can be verified are activities: (i) carried out with or (ii) witnessed by named persons; (iii) recorded on CCTV or photos; and (iv) that leave digital traces (e.g., phone calls) or physical traces (receipts).

VA has been frequently examined. A meta-analysis, including 14 studies, revealed that lie tellers indeed typically report fewer details that can be checked than truth tellers, *g* = 0.42 [93]. This effect becomes stronger when an Information Protocol is used: asking interviewees, where possible, to include details in their statement that the investigator can check. Truth tellers, more than lie tellers, report checkable details following such a request [93]. When an Information Protocol is employed, the difference between truth tellers and lie tellers in reporting verifiable details is large, *g* = 0.80.

Recently, Nisin and colleagues introduced the Context Embedded Perception (CEP) approach [99]. The core of CEP is that activities always take place at specific locations and at specific times. If lie tellers wish to avoid providing information that can be verified, they should avoid providing such contextual details. Initial research testing this CEP approach showed that the proportion of contextual details (contextual details / [contextual details + sensory details]) was indeed higher amongst truth tellers than amongst lie tellers [99]. 

### 5.3. Cognitive Credibility Assessment

One component of CCA is to help truth tellers to report as much information as possible by using specific interventions that facilitate reporting. Such so-called ‘encouraging interviewees to say more’ interventions should have a larger effect on truth tellers than on lie tellers because the truth tellers’ strategy to be forthcoming should result in more information than the lie tellers’ strategy to keep their stories simple. CCA encourages truth tellers to say more by addressing all three reasons mentioned earlier as to why truth tellers do not initially report all information they know, namely wrong expectations, lack of motivation, and inability to recall. 

Raising expectations about how much information someone is expected to report can be achieved through a Model Statement, which is an example of a detailed account of an event [100]. Researchers typically present it in an audiotaped format, but other formats are equally effective [101]. Exposing interviewees to a Model Statement leads to more information than an instruction to ‘provide as many details as possible’, probably because the Model Statement is a concrete example, which is easier to follow than (abstract) instructions [102]. 

Raising motivation to provide information can be achieved through a supportive second interviewer; someone who nods and smiles during an interview. A supportive interviewer makes the interview setting more comfortable, which facilitates truth teller’s reporting [103]. 

Facilitating recall of (visual) experiences can be achieved by asking interviewees to sketch whilst they narrate [104,105]. Sketching is a visual output, which matches visual memory better than a verbal output. Sketching also slows down the thinking process which gives interviewees more time to think about the event. 

Apart from the ‘encouraging interviewees to say more’ component, CCA has two more components: ‘imposing cognitive load’ and ‘asking unexpected questions’. The rationale behind imposing cognitive load is that research has shown that during interviews lie tellers experience more cognitive load than truth tellers [106,107,108]. Investigators can exploit this difference by making the interview setting more difficult. This should impair lie tellers more than truth tellers because the lie tellers’ cognitive resources are more depleted. Recent research supports this hypothesis [109]. Participants discussed their true opinions or false opinions about a societal issue (e.g., abortion, capital punishment, cannabis for personal use). In addition to this, half of the participants had to remember a car license plate at the same time. Larger speech differences between truth tellers and lie tellers occurred in this ‘secondary task’ condition than when they were just asked to discuss their opinion. 

Lie tellers prepare themselves for interviews by thinking about answers they could give to possible questions. The weakness in their planning is that they can never know which questions will be asked. Investigators could exploit this by asking a mixture of expected and unexpected questions. For truth tellers, it makes no difference in answering those two types of question. In contrast, since lie tellers can give their prepared answers to the expected questions but need to fabricate an answer to the unexpected questions, for them the unexpected questions are considerably more difficult to answer. Examples of unexpected questions are spatial questions [110] and questions about the planning of activities [111,112]. 

Recently, several CCA techniques and an adaptation of VA were combined into one interview protocol consisting of five phases [87]. The assumption was that truth tellers will report more additional details during each phase than lie tellers, due to truth tellers’ inclination to be forthcoming and lie tellers’ inclination to keep their stories simple. An initial free recall was followed by a Model Statement as an example for a second free recall. Then, a third free recall took place in Reverse Order (to impose cognitive load) followed by a fourth free recall in which interviewees spoke and sketched at the same time. In a final free recall, interviewees were asked to include, where possible, sources the investigator could check (named witnesses, CCTV footage, receipts etc.). Truth tellers reported more specific new details (complications and verifiable sources) during the subsequent phases than lie tellers. A complication is information that affects the storyteller and makes the recall more complicated (‘Initially we did not see each other because we were waiting at different entrances’). This criterion is an adaptation of CBCA-criterion 7: unexpected complications.

CCA has been widely researched to date. A meta-analysis of CCA research including 25 studies showed that using CCA techniques resulted in a moderate increase in differentiating between truth tellers and lie tellers (*d* = 0.42) compared with a standard condition [85]. A second meta-analysis included 23 studies in which observers attempted to detect deceit [113]. It revealed that observers were more accurate at distinguishing between truth tellers and lie tellers when a CCA technique was employed in the interview (60% accuracy) than when it was not employed (48% accuracy). CCA particularly increased accuracy rates when observers were knowledgeable about the verbal cues they should attend to. That is, knowledgeable observers obtained much higher accuracy (76%) than naïve observers (52%) [113]. 

### 5.4. Reality Interviewing

Reality Interviewing [88,89] is a standardised interview protocol consisting of five phases. It has the same assumption as CCA: truth tellers will report more additional details during each phase than lie tellers, due to their inclination to be forthcoming and lie tellers’ inclination to keep stories simple. Stage 1 (initial free recall stage) is an initial request to interviewees to report all they can remember about the alleged event. The initial free recall is followed by mnemonics, which are interview techniques that facilitate enhanced memory recall [114]. These mnemonics are derived from the Cognitive Interview [115,116,117], an interview protocol designed to elicit more information from cooperative witnesses. In Stage 2 (mental reinstatement of the context stage), participants are asked to report the event again, but this time to think about and include all associated details such as sights, sounds, smells, emotions, thoughts at the time, or anything else they remember from the time of the event. In Stage 3 (other perspective stage), participants are asked to recall the alleged event from another perspective: ‘If someone else had been present, what would they have seen?’ In Stage 4 (reverse order stage), participants are asked to report the event in reversed chronological order. In Stage 5 (final free recall stage), participants are asked for a final time to describe the alleged event in as much detail as possible. 

The Reality Interview also contains nine multiple choice questions (each with two alternatives) which are embedded in between the interview stages. For example, ‘If a police officer had been present, would they have noticed something wrong?’ These multiple-choice questions are included to force the participant to think deeper about the event. Answering ‘No’ would make it less likely that a follow-up question will be asked and thus be in alignment with lie tellers’ strategy to keep it simple.

Of the four interview protocols discussed in this section, RI is the least examined. All RI research examines the ability to detect truths and lies via the RI protocol as a whole without examining the diagnostic value of each of the five phases. However, we know from deception research that the Reverse Order instruction can facilitate lie detection [75,118]. There is no meta-analysis yet available examining the efficacy of RI. The results should be interpreted with some caution but the available studies to date showed that around 75% of truth tellers and lie tellers were correctly classified with the tool [119].

## 6. Comparing SUE, VA, CCA and RI

Table 2 summarises aspects of SUE, VA, CCA, and RI that we believe are important when comparing the four interview protocols. All protocols except SUE use the RM-list of criteria (sensory and contextual details) as dependent variables. In VA, a distinction is made between details that are verifiable and details that are not. More recently, VA examines the proportion of contextual details in a statement (contextual details / [contextual details + sensory details). CCA measures, apart from sensory and contextual details, complications (adapted from CBCA) and verifiable sources (adapted from VA). SUE is the only protocol that does not use RM coding, as it purely focuses on inconsistencies (within a statement or between the statement and available evidence).

All interview protocols are standardised in terms of procedure. An initial unprompted free recall is followed by specific questions and instructions. These questions and instructions are always the same in VA, CCA, and RI interviews but differ in SUE because they depend on the available evidence. However, the way to ask questions related to the evidence and how to strategically disclose the evidence is standardised in SUE (from weak source/low specificity questions to strong source/high specificity questions [91,96], like the standardised procedure of adaptive testing [120].

All four interview protocols received empirical support that they can successfully discriminate truth tellers from lie tellers, and only the support for RI is not based on a meta-analysis. Research into the four protocols is concentrated in a few labs (SUE: Granhag and Hartwig; VA: Nahari and Vrij; CCA: Vrij; and RI: Colwell). This is a limitation because we could have more faith in a verbal lie detection tool if researchers working in independent labs show that the tool works. 

It is difficult to compare the diagnostic value of the protocols but comparing the effect sizes may give us an estimate. Since effect sizes for RI do not exist, we only compare SUE, VA, and CCA. The results show that support for SUE is the strongest, and for CCA, the weakest. There appears to be a positive relationship between independent evidence available (available in SUE vs. potentially available in VA vs. not available in CCA) and diagnostic value. Someone could argue that, if the correct interview protocol is used, lie detection is somewhat easier when a statement can be compared with evidence than when this is not possible. However, another explanation for the superior effect sizes for SUE compared with CCA is possible. SUE experiments are specifically designed—the interviewer possesses evidence that can be used in a SUE interview. CCA are typically not specifically designed for CCA assessments. That is, interviewees report self-generated stories about an alleged trip they made or about a memorable event [121]. If these events only contained a few complications, CCA assessments are unlikely to reveal substantial veracity effects. If researchers would construct events that include many complications truth tellers could report, CCA assessments could become more diagnostic. 

Cross-cultural research testing the efficacy of the four interview protocols is scarce and we know only one such experiment. In [122], researchers tested the efficacy of the Model Statement technique in Russian and South Korean participants and found that it was effective in eliciting complications as a cue to truthfulness. The same finding was also obtained in UK participants [102], suggesting that the Model Statement technique works cross-culturally. 

All four interview protocols are used by practitioners, including police and intelligence services, but its use is limited. There are at least two reasons for this. First, there is not much opportunity to learn the protocols because commercial training programs are not available. Second, for these protocols to work, interviewers should ask just a few open-ended questions, and provide many opportunities for interviewees to talk. This is not how interviewers typically conduct interviews. Interviewers often interrupt interviewees [123,124] and ask many questions to which interviewees only give short answers [125,126]. The discrepancy between a typically conducted interview and an interview suited for using the four interview protocols makes it difficult to teach the protocols to practitioners. Not only do they need to learn how to use the four protocols, they also need to abandon their old habit of asking so many questions. Third, some of the instructions may limit its use. For example, in the United Kingdom, defence lawyers will object against the Other Perspective Stage because it invites suspects to discuss something hypothetical (what would another person have experienced). 

The underlying rationale of all four interview protocols Is that truth tellers are willing to be forthcoming. They also assume that truth tellers do not report all information they know spontaneously, and that specific questions or instructions are needed to achieve this. CCA and RI put more effort than SUE and VA into encouraging truth tellers to report all they know by using memory-enhancement techniques (e.g., sketching, context reinstatement). CCA goes one step further compared with RI. CCA also includes instructions to: (i) raise expectations amongst truth tellers about how much information they are expected to report (e.g., Model Statement) and (ii) motivate truth tellers to report more information (e.g., supportive interviewer). This suggests that CCA elicits more information than RI, which was indeed found in the only experiment to date in which the two protocols were compared [65].

The underlying rationale of SUE and VA is that lie tellers strategically avoid reporting incriminating information. Since evidence is available to the interviewer in SUE or potentially available in VA (e.g., truth tellers may be able to report evidence), these interview protocols are designed to exploit differences between truth tellers and lie tellers in being forthcoming or evasive in reporting critical evidence. The underlying rationale of CCA and RI is that lie tellers prefer to keep their stories simple. Since evidence is unavailable when CCA and RI are used, these protocols are designed to exploit differences between truth tellers and lie tellers in being forthcoming or reluctant when reporting information in general. 

Which interview protocol to use depends on the available evidence. If interviewers possess independence evidence about the event under investigation (e.g., witnesses, CCTV footage, credit card receipts), SUE may be the preferred method. If evidence is potentially available (truth tellers will be able to provide evidence when asked to do so), VA may be the preferred method. If interviewers do not possess evidence and truth tellers are not able to provide such evidence, CCA and RI are the preferred methods. In police interviews, investigators often possess evidence. In other scenarios, the situation could be different. In situations where evidence is not available or cannot be revealed to the suspect, investigators can ask interviewees to provide such evidence. However, investigators can be reluctant to ask interviewees to provide evidence because interviewees could interpret this as an indication that the evidence against them is weak. If interviewees think that the evidence against them is weak, they may decide to ‘shut down’ [127,128] which will impair the interviewers’ ability to gather information and to detect deceit. It is also possible that truth tellers cannot provide verifiable information about key elements of their activities. For example, a source sent on a mission could be able to provide verifiable evidence about the location he went to but may be unable to demonstrate what he did there (key element) when CCTV cameras and witnesses are unavailable. 

Note that the recommendation for when to use which tool could be more complex than suggested in the previous paragraph. For example, if, on the one hand, the interviewer has little and particularly weak evidence regarding the event, and on the other hand, it is an event that included a lot of complications, it could be recommended to use CCA rather than SUE.

## 7. The Future of Verbal Lie Detection

In recent years we have suggested several venues for future verbal lie detection research [129,130]. This included researching verbal lie detection: (i) in cross-cultural settings, (ii) in different deception scenarios (vetting scenarios, lying about intentions, lying through omitting information), and (iii) when using interpreters. We recommended searching for new verbal cues, particularly those that indicate deceit (lie tellers report the cue more than truth tellers) rather than truthfulness (truth tellers report the cue more than lie tellers). We also suggested research examining the efficacy of countermeasures (can lie tellers sound like truth tellers when they are aware of how the interview protocols work?), and suggested research aimed at further developing interview protocols that use within-subjects comparisons (comparing different responses made by the same interviewee in a single interview). For more information about these ideas, see [129,130].

In this section, we will outline some ideas about how insight into truth tellers’ and lie tellers’ brain activity could help verbal lie detection. To date, neuroscience-based research on verbal lie detection has focused on measuring brain activity to simple responses given by individuals who have been asked single questions [131]. This is a much more basic interview protocol than used in real life where often a single verbal response could raise suspicion in an interviewer which then could lead to follow-up questions to home in onto this potential lie. This more interactive interviewing approach is almost impossible with neuroimaging, in part because the signal-to-noise ratio of ongoing brain signals for complex cognitive processes such as lying, especially in interactive settings, is much smaller than that of overt signals such as verbal responses. That is, the neural signatures associated with a specific verbal response would be buried in neural noise produced by everything else that occurs in the interviewee’s brain during that event and could not be used to guide the formulation of further questions. Reducing such noise would require combining information from several tens of similar events over extended periods of time. In addition, there is the practical problem of having to get access to and to use MRI machines each time to conduct an interview. However, there are more ways in which the earlier discussed ideas about interviewing to detect deception could benefit from neuroscience techniques (e.g., functional magnetic resonance imaging, functional near infrared spectroscopy, electroencephalography, transcranial magnetic stimulation). These ways are indirect. That is, they provide further insight into verbal lie detection and this insight could further advance the development of verbal lie detection tools. Below are a few suggestions.

First, meta-analyses of brain imaging studies of deception have generally supported the idea that deception is associated with an increase in cognitive control compared with truth telling, as suggested by stronger ventrolateral, dorsolateral, and medial prefrontal cortical engagement [131,132,133]. Precise information about the activation patterns in these brain regions during tasks that require cognitive control could potentially be used to enhance verbal lie detection methods. Neuroimaging data could be used to try to maximise interference effects during an interview by figuring out which secondary tasks generate brain activation patterns that overlap the most with those produced when generating lies. Indeed, there is evidence that the degree of overlap between the neural patterns elicited by two tasks predicts the degree of behavioural interference between them. For example, [134] found that the more similar the pattern of brain activity elicited by two tasks (e.g., 2-back and tone counting), the lower the accuracy when the two tasks were performed simultaneously. 

Second, instead of indirectly interfering with brain regions involved in generating lies with a secondary task, a more direct approach to potentially boost lie detection would be to transiently deactivate brain regions involved in producing lies using non-invasive brain stimulation methods such as transcranial magnetic stimulation [135]. For example, one could use Theta Burst Stimulation [136] to temporarily disrupt function in one or more brain regions that routinely show up in brain imaging studies as being activated when lying and then administer an interviewing to detect deception interview protocol. Brain stimulation effects could greatly amplify the verbal veracity differences because lie tellers should find it much more difficult (or impossible) to generate lies. Of course, special attention to neuroethical issues is needed when using these methods in a lie detection context.

Third, brain activity could also be compared when interviewees answer expected and unexpected questions, assuming that a sufficiently large number of such questions can be asked during the interview. Unexpected questions should surprise truth tellers and lie tellers in equal measure. However, it should be more difficult for lie tellers to answer the unexpected questions than the expected questions, because they can give their planned answers to the expected questions but must produce spontaneous answers to the unexpected questions. This difference in difficulty could become apparent in lie tellers’ brain activity, as already documented when comparing brain activation patterns during memorised and spontaneous lying [137]. In contrast, for truth tellers, the difference in difficulty in answering the expected and unexpected questions should be less pronounced than in lie tellers, which could also become apparent in their brain activity.

In addition, when comparing the brain activity of truth tellers and lie tellers in answering the unexpected questions, there should be greater brain activation in brain networks involved in episodic memory retrieval in truth tellers (because they will think back about the event), whereas in lie tellers, there should be greater engagement of brain networks involved in creative cognition (because they will make up an answer) [138]. 

Fourth, brain activation data could also be useful with the Model Statement technique. Listening to a Model Statement makes both truth tellers and lie tellers realise that they are expected to provide additional details. When realising this, truth tellers could continue listening to the Model Statement because the additional information they are going to report is already available to them. In contrast, lie tellers must start thinking about possible extra details they must report. This will shift attention away from listening to the Model Statement because they will be unable to think about what to say next and listen to the Model Statement at the same time. Measuring brain activity while interviewees are engaged in listening to a Model Statement could test this hypothesis. We expect that brain areas related to listening and verbal decoding should be more engaged in truth tellers than in lie tellers because of truth tellers’ enhanced listening to the Model Statement [139]. Brain regions involved in creative cognition should be more engaged in lie tellers than in truth tellers because of lie tellers’ need to generate additional details [138].

Fifth, cognitive neuroscientists could compare truth tellers’ and lie tellers’ brain activity when they report an event that supposedly happened a long time ago (i.e., years ago). 

Truth tellers should retrieve remote autobiographical memories (when the events of interest took place), whereas lie tellers should retrieve more recent autobiographical memories (when they planned what to say during the interview). These two types of memories elicit different patterns of activation in brain regions, such as the ventromedial prefrontal cortex and the hippocampus [140], and these differences could be used to help decide whether the interviewee is engaged in lying or telling the truth. A similar pattern of results could emerge when interviewees are repeatedly interviewed about the same event. In those situations, truth tellers tend to think about the event again [141]. In contrast, lie tellers are typically concerned about consistency [142] and will think about what they have said in the previous interview [141]. Repeated interviewing should therefore, in truth tellers, activate brain areas associated with remote autobiographical memories (when the event occurred) and, in lie tellers, should activate brain areas associated with more recent autobiographical memories (e.g., the time of their first interview). The same finding could also emerge when brain activity is examined in a reverse order interview when an initial free recall is followed by the request to report the event in reverse order. Truth tellers are expected to think about the event again when recalling their experiences in reverse order, whereas lie tellers will be inclined to think about what they just said so that they can repeat themselves in reverse order. 

Sixth, brain activity could also be examined using the sketching method. The request to sketch whilst recalling the event should encourage truth tellers to visualise their experiences and hence engage brain regions involved in visual mental imagery and episodic memory retrieval [143,144]. In contrast, lie tellers reporting something they have not experienced but only fabricated, often on the fly, may show increased engagement of brain regions involved in semantic memory representation and reactivation [145]. Sometimes lie tellers will use content from real experiences to design their lies. These could be detected if the lie teller uses an existing visual memory of a real event that they experienced much earlier or much later than the alleged event, as we described in the previous paragraph.

Although these neuroscience-based methods look promising, it is important to keep in mind that a critical issue for their applications is whether they provide reliable information at the level of the individual person. In other words, even if certain neural differences are measurable when averaging data over a group, they may be too small to be diagnostic at the single individual level, at least with current technology and paradigms. Determining this will require interdisciplinary work and systematic collaborative work between neuroscientists, verbal lie detection researchers, and practitioners.

## Figures and Tables

**Table 1:** A comparison between the CBCA, RM, and SCAN tools.

	CBCA	RM	SCAN
Do the methods use clusters of variables?	Yes	Yes	Yes
Is the method standardised?	Yes	Yes	No
Is there empirical support for the method?	Yes, *d* = 0.78 in children and *d* = 0.55 in adults	Yes, *d* = 0.55 (adults only)	No
Is the method widely researched across labs?	Yes	Yes	Yes
Is the method tested in non-WEIRD cultures?	Probably not often	Probably not often	Probably not often
Is it used by practitioners?	Yes	No	Yes
Is the method easy to learn and use for practitioners?	No	Yes	No
Is an underlying rationale provided why the method should work?	Yes. Lie tellers are unable and unwilling to report as many details as truth tellers	Yes. Truth tellers’ memory differs from lie teller’s memory	No
Does the method involve an interview protocol to elicit differences between truth tellers and lie tellers?	No	No	No

Note: WEIRD: Western, educated, industrialised, rich, and democratic.

**Table 2:** A comparison between SUE, VA, CCA, and RI.

	SUE	VA	CCA	RI
Do the methods use clusters of variables?	No	Yes	Yes	Yes
Is the method standardised?	Yes in terms of procedure	Yes in terms of procedure and questions asked	Yes in terms of procedure and questions asked	Yes in terms of procedure and questions asked
Is there empirical support for the method?	*d* = 1.06 (statement – evidence consistency)	g = 0.42 (verifiable details) g = 0.80 (verifiable details after Information Protocol is implemented)	*d* = 42 (comparing CCA conditions with control conditions) Observers: 48% accuracy in standard interviews and 60% in CCA interviews (76% if observers are knowledgeable about cues)	75% accuracy
Is the method widely researched across labs?	Dominated by Granhag and Hartwig and colleagues	Dominated by Nahari and Vrij and colleagues	Dominated by Vrij and colleagues	Dominated by Colwell and colleagues
Is independent evidence necessary?	Yes	Yes	No	No
Is the method tested in non-WEIRD cultures?	No	No	Yes, part of it	No
Is it used by practitioners?	Yes	Yes	Yes	Yes
Is the method easy to learn and use for practitioners?	No	No	No	No
Is an underlying rationale provided why the method should work?	Truth tellers: Be forthcoming *Lie tellers*: Avoid reporting incriminating evidence	Truth tellers: Be forthcoming *Lie tellers*: Avoid reporting incriminating evidence	Truth tellers: Be forthcoming *Lie tellers*: Keep stories simple	Truth tellers: Be forthcoming *Lie tellers*: Keep stories simple
Can it be used all the time?	Only when evidence is available	Only when evidence is potentially available	Yes	Yes
When should it be used?	When evidence is available	When evidence is potentially available	When no evidence can be obtained	When no evidence can be obtained

Note: WEIRD: Western, educated, industrialised, rich, and democratic.

## Data Availability

Not applicable.
